# Further karyosystematic studies of the *Boreonectes griseostriatus* (De Geer) group of sibling species (Coleoptera, Dytiscidae)–characterisation of *B. emmerichi* (Falkenström, 1936) and additional European data

**DOI:** 10.3897/CompCytogen.v9i1.4463

**Published:** 2015-03-31

**Authors:** Robert B. Angus, Elizabeth M. Angus, Fenglong Jia, Zhen-ning Chen, Ying Zhang

**Affiliations:** 1 Department of Life Sciences (Entomology), The Natural History Museum, Cromwell Road, London SW7 5BD, UK; 2 Biomedical Imaging Unit, Level B South Block, Mail point 12, General Hospital, Southampton SO16 6YD, UK; 3 Institute of Entomology, Life Science School, Sun Yat-sen University, Guangzhou, 510275, Guangdong, China; 4 Biology and Geography School, Qinghai Normal University, Wusi West Road 38#, 81000, Xining, Qinghai Province, China

**Keywords:** Coleoptera, Dytiscidae, Karyotype, C-banding, species complex, Tibet, lectotype, Sikkim, first record from India

## Abstract

A lectotype is designated for the Tibetan species *Deronectes emmerichi* Falkenström, 1936 (Currently *Boreonectes emmerichi* (Falkenström)), and its habitus, as well as the median lobe and parameres of its aedeagus, are figured along with additional comparative material. Material of *Boreonectes emmerichi* from Sikkim (BMNH) represents the first record of a *Boreonectes* Angus, 2010 species from India. The karyotype of *Boreonectes emmerichi* is described as having 26 pairs of autosomes plus sex chromosomes which are X0 (♂), XX (♀). The karyotype is most like that of *Boreonectes macedonicus* (Géuorguiev, 1959), but with slight differences. Additional chromosomal information is given for *Boreonectes griseostriatus griseostriatus* (De Geer, 1774) in the French Alps, *Boreonectes griseostriatus strandi* (Brinck, 1943) on the Kola Peninsula, *Boreonectes multilineatus* (Falkenström, 1922) in the Pyrenees and *Boreonectes ibericus* (Dutton & Angus, 2007) in the Spanish Picos de Europa.

## Introduction

The group of species related to *Boreonectes griseostriatus* (De Geer, 1774) presents serious taxonomic problems. Helliesen (1890), working in Norway, recognised an inland montane stripy form, which he regarded as true *griseostriatus*, and a coastal, more blotchy form, which he named *maritimus* sp. n. Falkenström (1922) showed that De Geer’s *griseostriatus* in fact referred to the coastal form, and described the inland form as *Deronectes multilineatus* Falkenström, 1922. Many subsequent authors rejected the notion that more than one species was involved. Thus Zaitzev (1953) in the Israeli English translation (1972) noted that “This species (*Boreonectes griseostriatus*) varies markedly in many characters; all attempts to establish subspecies and varieties are unjustified, because almost all varieties are connected by transitions.” However, chromosome analysis by Angus began to show that several species were involved and Dutton and Angus (2007) demonstrated the existence of seven chromosomally distinct species, all difficult or even impossible to recognise on external morphology. Further investigations by Angus (2008, 2009, 2010a, b) gave more information, culminating in the erection of a new genus, *Boreonectes* Angus, 2010, for these and some related species. However, there remain a number of described Palaearctic taxa for which there are no chromosomal data. Prominent among these is *Boreonectes emmerichi* (Falkenström, 1936), described from Sichuan and regarded as widespread in Tibet (Nilsson and Hájek 2015).

A beetle-collecting trip to the Tibetan Plateau in June 2013 (Angus 2013) enabled the senior author to obtain living material of *Boreonectes emmerichi*. Laboratory facilities in Qinghai Normal University enabled preparation of slides with dividing cells for karyotype investigation. The opportunity is also taken to include additional data on European species.

## Material and methods

The species studied are listed in Table 1 and the localities are shown in Figs 4, 7–9. The museums housing material discussed here are the Natural History Museum, London (BMNH), the Naturhistoriska Riksmuseet, Stockholm (NRMS), the Museum of Biology, Sun Yat-sen University, Guangzhou (SYSU) and the Barcelona branch of the Spanish Institute for Evolutionary Biology (IBEB). The methods of chromosome preparation, C-banding and photography are as given by Dutton and Angus (2007), with the treatment times in colchicine and ½-isotonic KCl each 12 minutes. With the Chinese material (*Boreonectes emmerichi*) C-banding was attempted in London, several weeks after initial preparation of the slides. This was moderately successful, though not as good as that obtained with 48-hour-old slides. Specimens from which chromosomes were obtained are housed in the BMNH. Habitus photographs of whole beetles (apart from the Nam Tso specimen, for which Ignacio Ribera sent the picture) were taken with a Leica M125 stereomicroscope + Canon EOS 550D digital camera, and the parameres with a Zeiss Axioskop + Canon EOS 450D digital camera, in the Sackler Bioimaging Laboratory of the Natural History Museum. Both were stacked using Helicon Focus software. The scanning electron micrographs are of uncoated specimens. Those shown in Fig. 2a–f were taken in the Electron Microscope Unit of the Natural History Museum, using a Zeiss Leo 1455VP SEM in low vacuum mode and back-scattered electrons. The one in Fig. 2g was taken in the Biomedical Imaging Unit of Southampton General Hospital, using an FEI Quanta 200 SEM in low vacuum mode, using secondary electrons to image.

**Table 1.** Material used for chromosome analysis.

Species	Locality	No. of specimens analysed	Location of specimens
*Boreonectes emmerichi*	China, Qinghai Province, Gangca, 1 km SE of Gangca Dasi. (Fig. 4)	2 ♂♂, 1♀ (Whole beetle illustrated.)	BMNH
	China, Qinghai Province, ca 20 km W of Maduo. (Fig. 4)	3♂♂, 2♀♀ (Whole beetles illustrated.)	BMNH
	CHINA, Xizang Autonomous Region, Sejilashan. (Fig. 4)	(Whole beetle illustrated.)	SYSU, BMNH
	CHINA, Xizang Autonomous Region, Nam Tso. (Fig. 4)	(Whole beetle illustrated.)	IBEB
	INDIA, Sikkim, Lachen. (Fig. 4)	(Whole beetle illustrated.)	BMNH
*Boreonectes griseostriatus griseostriatus*	France, Savoie, S of Lac du Mont Cenis. (Fig. 7)	2 ♂♂, 2♀♀	BMNH
*Boreonectes griseostriatus strandi*	Russia, Kola Peninsula, near Teriberka. Leg. P. Petrov. (Fig. 8)	5 ♂♂, 4 ♀♀	BMNH
*Boreonectes multilineatus*	France, Hautes-Pyrénées, Lac d’Anapéou. Leg. F. Bameul. (Fig. 9)	5 ♂♂	BMNH
*Boreonectes ibericus*	Spain, Cantabria, Lagos de Lloroza. (Fig. 9)	1 ♂	BMNH

## Results and discussion

### *Boreonectes emmerichi* (Falkenström, 1936: 88).

**Figure 1.**
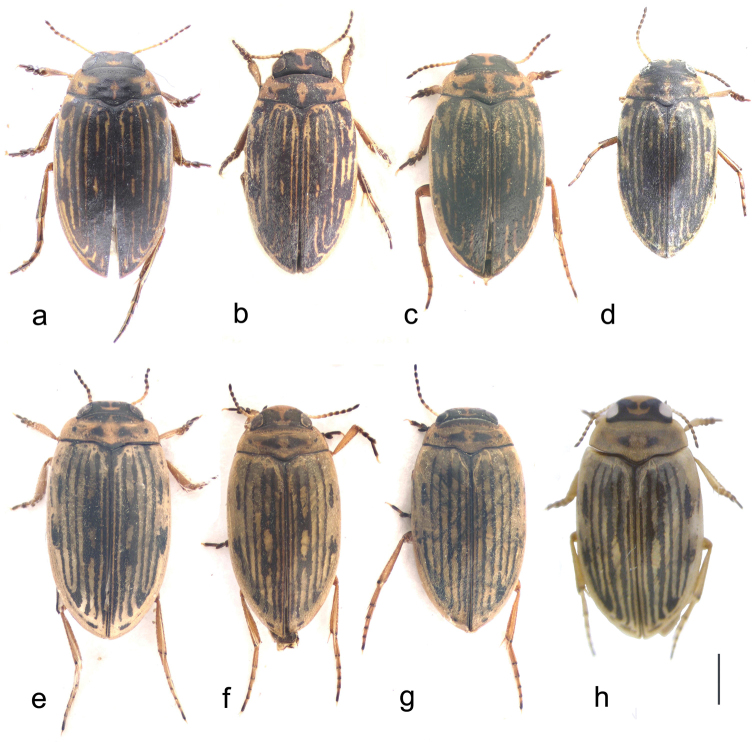
Habitus photographs of *Boreonectes emmerichi*. **a** lectotype ♂ **b** paralectotype ♀ **c** dark ♂, Sejilashan, Xizang **d** small dark ♂; Lachen, Sikkim **e** ♀, Gangca Dasi **f** ♂, Maduo (aedeagal median lobe: Fig. 2c) **g** ♂, Maduo (aedeagal median lobe: Fig. 2b) **h** ♀, Nam Tso. Scale = 1 mm.

**Figure 2.**
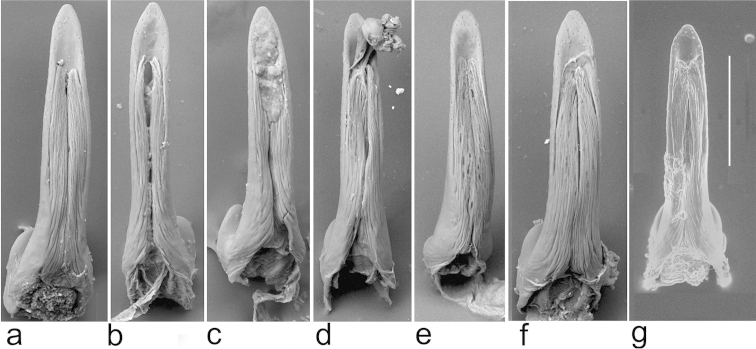
Scanning electron micrographs of aedeagal median lobes (ventral view). **a** *Boreonectes emmerichi*, lectotype **b–d** *Boreonectes emmerichi*, Maduo **d** with a partially extruded spermatophore **e**, **f** *Boreonectes emmerichi*, Gangca Dasi **g** *Boreonectes macedonicus*, Karanikoličko Jezero, Macedonia; Scale = 0.2 mm.

**Figure 3.**
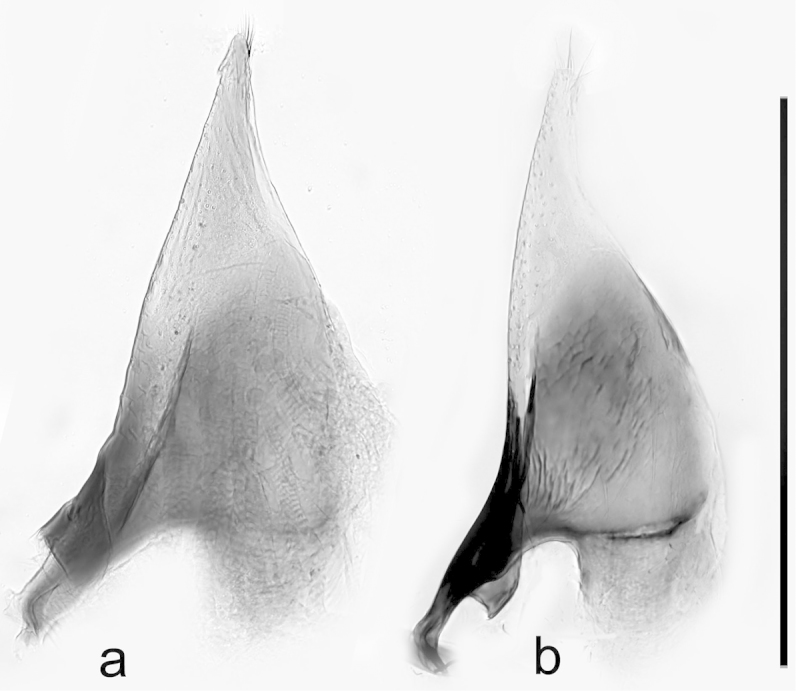
*Boreonectes emmerichi*, left parameres. **a** lectotype **b** Gangca Dasi. Scale = 0.5 mm.

Falkenström described *Boreonectes emmerichi* (as *Deronectes emmerichi*) from 15 specimens, including five males, from the Kangding area of Sichuan, on the eastern edge of the Tibetan Plateau. Seven specimens are listed with the data “China, Szechuan, Mukue-Tatsienlu” and eight “China, Szechuan, Tatsienlu Tjiji (Urwald Rodungen)”. Tatsienlu is the former name of Kangding and Urwald Rodungen (a German term) are clearings in primary forest. Three syntypes, an intact male labelled as Holotypus, a dissected male without the genitalia and a female labelled as Allotypus, are housed in the Falkenström collection (NRMS) and there is a further female syntype in London (BMNH). We do not know the whereabouts of the other specimens listed by Falkenström. The female with the Allotypus label has a data label “China, Szechuan, Mukue-Tatsienlu” but all the others have the labels “China, Szechuan, Tatsienlu Tjiji (Urwald Rodungen)”. The intact male is here designated lectotype, so the type locality is fixed as Tatsienlu Tjiji, 29°59.906'N, 101°57.492'E. The remaining specimens are paralectotypes. We have dissected this male, and the median lobe (penis) is shown in Fig. 2a, left paramere in Fig. 3a and habitus in Fig. 1a. As noted by Falkenström, this is a rather dark species, with the black markings very heavy. All the specimens are similarly dark, and the London female is shown in Fig. 1b. This heavy dark pattern is matched by material collected by Fenglong Jia in swampy pools among dense bushes on Sejilashan Mountain near Namcha Barwa in SE Xizang (Jia et al. 2012) (Fig. 1c). It seems likely that this dark pattern is associated with wooded or bushy habitats–the type localities are clearly in a wooded zone. Material from Sikkim (BMNH) is also dark, and rather small (Fig. 1d). This material, 10 specimens with the data “Sikkim. Tangu. 11500 ft. 26.iv.1924. Maj. R. W. G. Hingston” represents the first known occurrence of a *Boreonectes* species in India. The locality Tangu is given by Google earth as Lachen, with an altitude of 2749 m. To attain an altitude of 11500 ft (3505 m) it is necessary to travel about 10 km further north along the road to Tibet (Gurudongmar Road). One specimen is further labelled “In a mountain pool” and one female is carrying a spermatophore of the *Nebrioporus* pattern (Shirt and Angus 1992).

Material from more open areas of the Tibetan Plateau, from Gangca in the north to Nam Tso in the south, has the dark markings less extensive, especially on the pronotum (Fig. 1e–h). There is some variation in the size and shape of the median lobe. The lectotype has the median lobe elongate and slightly longer than in some material (Fig. 2a), but material taken in shallow pools in the riverine flatlands about 20 km E of Maduo includes specimens with larger more elongate median lobes (Fig. 1b) as well as shorter relatively broader ones (Fig. 1c, d) while material from Gangca Dasi shows some variation in median lobe width (Fig. 1e, f). This material appears to be chromosomally uniform, as would be expected if all the specimens belong to the same species, *Boreonectes emmerichi*. The median lobe of *Boreonectes macedonicus*, which has a similar karyotype to *Boreonectes emmerichi*, is shown in Fig. 2g. It is noticeably smaller than that of *Boreonectes emmerichi*. Fig. 3a shows the left paramere of the *Boreonectes emmerichi* lectotype, while a Gangca Dasi specimen is shown in Fig. 3b.

**Figure 4.**
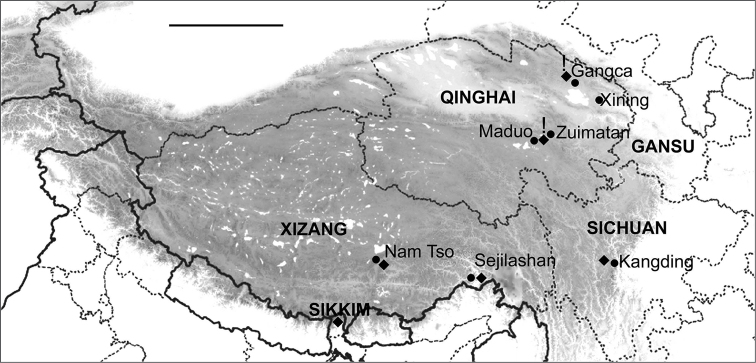
Map of the Tibetan Plateau showing *Boreonectes emmerichi* localities (◆). Localities from which specimens giving chromosome preparations were obtained indicated by exclamation mark (!). Scale line = 500 km.

Further support for the view that all the Tibetan material discussed here belongs to the same species is given by unpublished preliminary DNA data supplied by Ignacio Ribera. The mitochondrial gene CO1 is very similar in material from Gangca, Maduo and Nam Tso (S Tibet 21.VII.10, S Namtso lake 4750m, banks, 30°37'03"N, 90°43'30"E, leg. Joachim Schmidt) with slight differences (less than 1.6% overall) which correspond with geographical distance between the populations (Fig. 4), and show a considerably larger separation from any other *Boreonectes* species for which there are molecular data.

**Figure 5.**
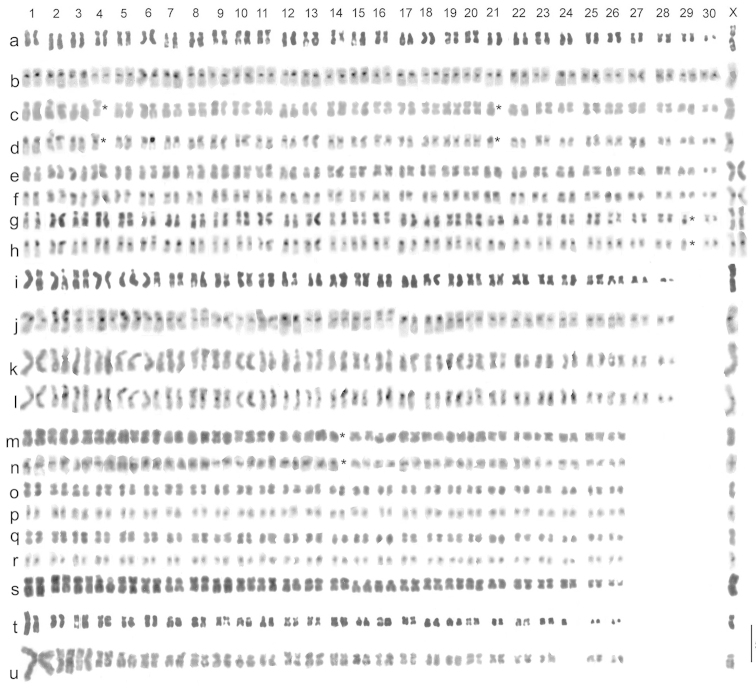
Mitotic chromosomes of *Boreonectes* spp., arranged as karyotypes. **a–d** *Boreonectes griseostriatus* **a, b** Sweden (from Dutton, Angus (2007)) **c, d** Mt Cenis **e–h** *Boreonectes griseostriatus strandi*, Teriberka **i–l** *Boreonectes multilineatus* **i, j** Scotland (from Dutton, Angus (2007)) **k, l** Lac d’Anapéou **m–r** *Boreonectes emmerichi* **m, n** from mid-gut, Maduo **o–r** from testis, Gangca Dasi **s** *Boreonectes macedonicus*, Crno Ezero, Macedonia (from Angus (2008)) **t, u** *Boreonectes ibericus* **t** Peña Lara (from Dutton, Angus (2007)) **u** Lagos de Lloroza. **b, d, f, h, j, l, n, p, r** C-banded, the rest plain, Giemsa-stained. Missing chromosomes indicated by asterisks (*). Scale line = 5 μm.

**Figure 6.**
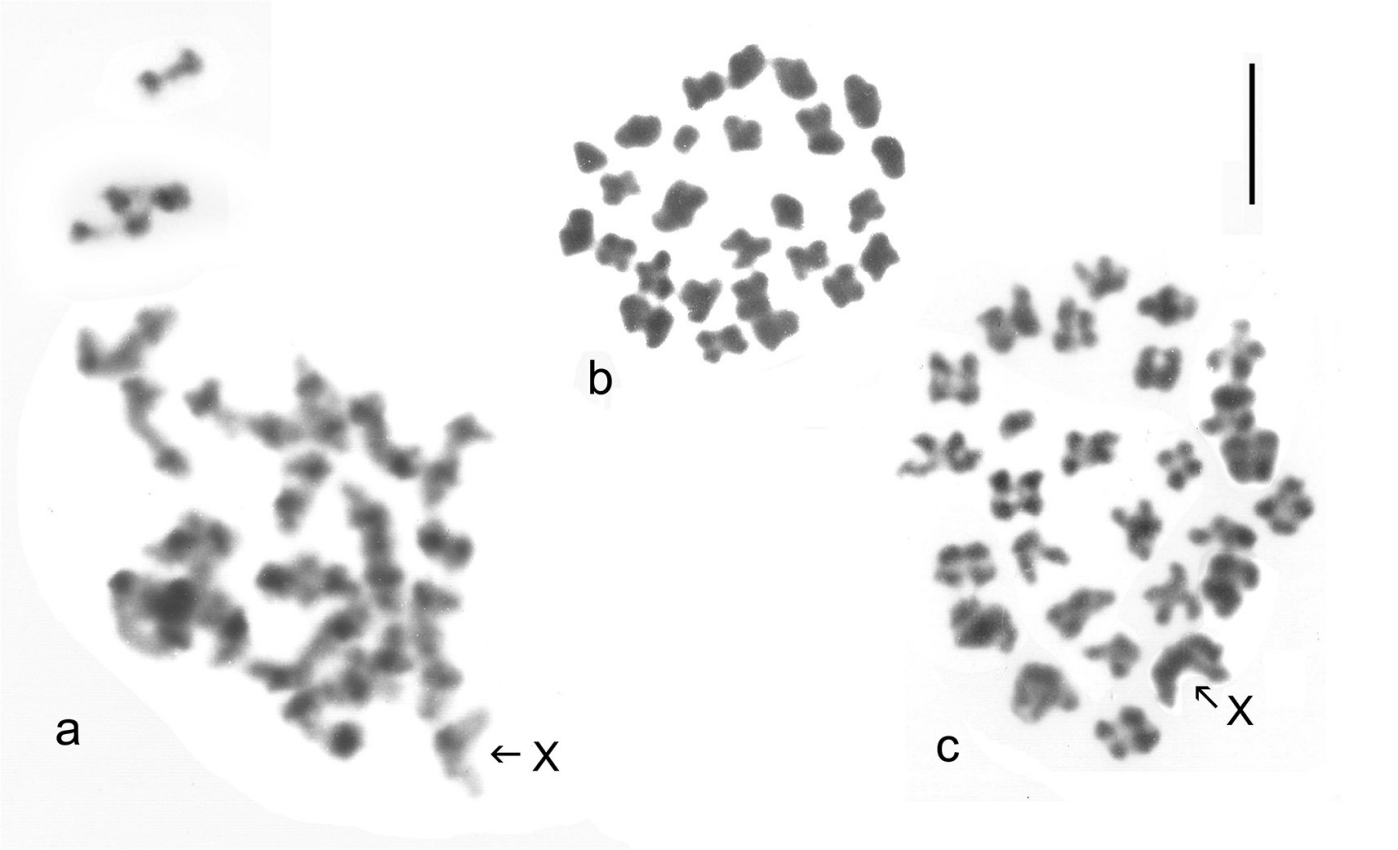
Meiotic chromosomes of *Boreonectes emmerichi* from Gangca Dasi. **a** metaphase I, C-banded **b**, **c** metaphase II plain, Giemsa-stained **b** male-determining nucleus with 26 chromosomes **c** female-determining nucleus with 27 chromosomes including X. Scale line = 5 μm.

Mitotic chromosomes, arranged as karyotypes, are shown in Fig. 5m–r, and meiotic chromosomes in Fig. 6. There are 26 pairs of autosomes and the sex chromosomes are X0 (♂), XX (♀). This karyotype closely resembles that of *Boreonectes macedonicus* (Fig. 5s), but autosome 12 appears slightly more evenly metacentric and autosome 26 appears more or less the same size as pairs 24 and 25, as against clearly smaller than these pairs in *Boreonectes macedonicus*. The X chromosome is shown as an unpaired submetacentric in the C-banded first metaphase of meiosis shown in Fig. 6a. In the mitotic karyotypes the X chromosome appears about the same size as autosome 1 (longer than autosome 1 in *Boreonectes macedonicus*) but in second metaphase of meiosis (Fig. 6c) it appears longer. These are difficult karyotypes to work with. The chromosomes appear very condensed in the few successful preparations obtained, and the C-banding is not very good. Nevertheless, these karyotypes are sufficient to show that this Tibetan material has its own characteristic karyotype, and the differences in the relative lengths of autosomes 24, 25 and 26 when compared with those of *Boreonectes macedonicus* are sufficient to demonstrate that there has been translocation of material between different autosomes, indicating that these are indeed different species.

### *Boreonectes griseostriatus griseostriatus* (De Geer, 1774)

**Figure 7.**
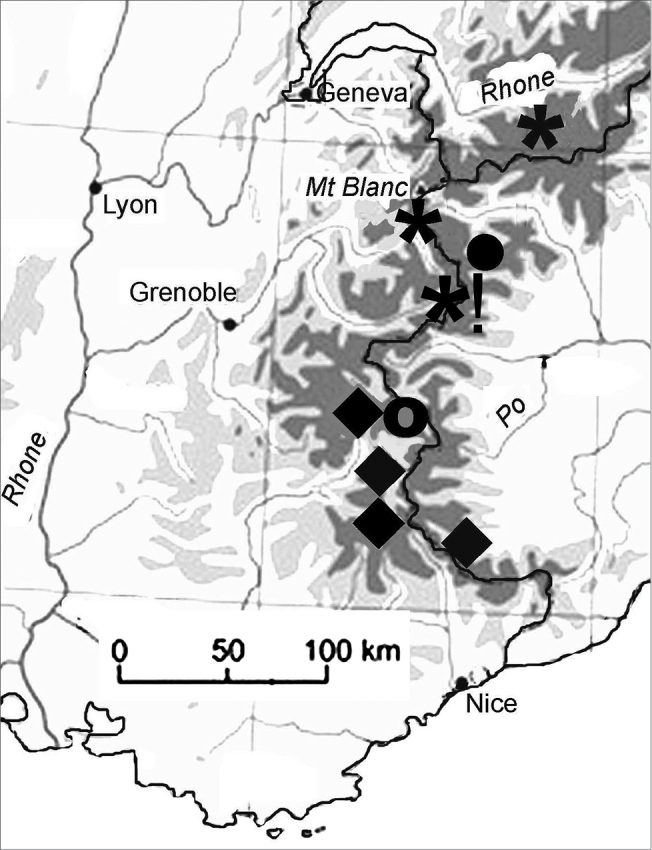
Map of the western Alps showing the sources of populations of *Boreonectes griseostriatus* and relatives from which chromosome data were obtained. Symbols: * *griseostriatus* • *alpestris* ◆ *ibericus* ○ *inexpectatus*. New data indicated by exclamation mark (!).

Mitotic chromosomes of a Mt Cenis specimen, arranged as a karyotype are shown in Fig. 5c (plain, Giemsa stained) and d (C-banded), while Fig. 5a, b shows Swedish material for comparison. Although two chromosomes are missing from the Mt Cenis karyotype (positions marked with asterisks (*) in the figure), the forms of the remaining chromosomes make it clear that this is indeed *Boreonectes griseostriatus*. The localities in the western Alps from which *Boreonectes* populations yielding karyotypes have been obtained are shown in Fig. 7. The presence of *Boreonectes griseostriatus* in the Mt Cenis area suggests that the range of *Boreonectes alpestris* (Dutton & Angus, 2007) (nearest locality: Italy, Colle del Nivolet in the Gran Paradiso (Angus 2010b)) may not extend into France.

### *Boreonectes griseostriatus strandi* (Brinck, 1943)

Mitotic chromosomes of a Teriberka specimen, arranged as karyotypes, are shown in Fig. 5e, g (plain, Giemsa stained) and Fig. 5f, h (C-banded). The karyotype shown in Fig. 5g, h lacks one replicate of autosome 29 but is included as the form of the chromosomes is particularly clear. As expected of *Boreonectes griseostriatus strandi*, the chromosomes show no differences from those of typical *Boreonectes griseostriatus* (Fig. 5a–d). The distinguishing feature of *Boreonectes griseostriatus strandi* is its size. Angus (2009) gave the size range of Norwegian *strandi* as 4.6–5.2 mm (♂), 4.6–5.3 mm (♀) and Brinck gave the size range of *strandi* (both sexes) as 4.8–5.5 mm as against 4.0–4.8 mm for normal *griseostriatus*. The size range of the Teriberka material is 5.0–5.1 mm (♂), 4.9–5.2 mm (♀), clearly *strandi*.

**Figure 8.**
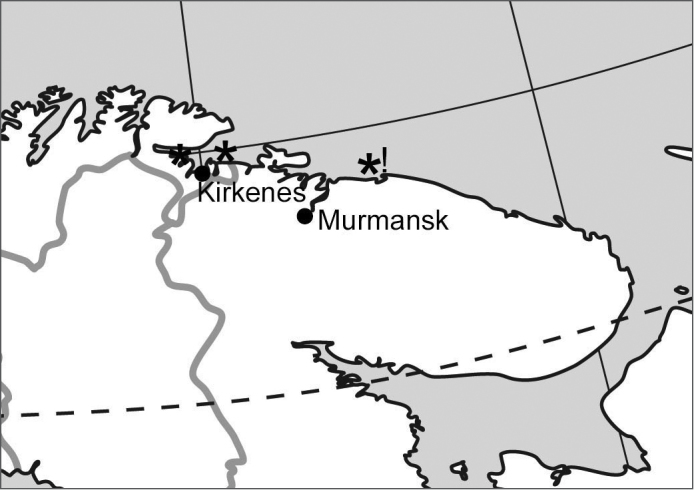
Map showing the localities of *Boreonectes griseostriatus strandi* populations from which chromosome data were obtained (*). New record indicated by exclamation mark (!).

The localities from which populations of *Boreonectes griseostriatus strandi* yielding karyotypes have been obtained are shown in Fig. 8. The most easterly published record of *Boreonectes griseostriatus strandi* is from near Murmansk (Brinck 1943, Lindberg 1930), about 70 km west of Teriberka.

It should be noted that Nilsson and Hájek (2015) list *strandi* as a straight synonym of *griseostriatus*. Here we follow Brinck (1943) in regarding it as a subspecies in view of its larger size and restricted geographical distribution.

### *Boreonectes multilineatus* (Falkenström, 1922)

**Figure 9.**
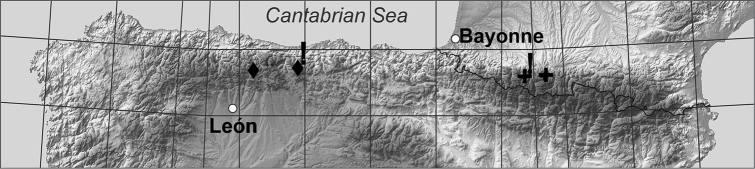
Map showing the localities of *Boreonectes multilineatus* populations in the Pyrenees (**+**), and of *Boreonectes ibericus* populations in the Picos de Europa area (◆), from which chromosome data were obtained. New data indicated by exclamation mark (!).

Mitotic chromosomes of a specimen from the Lac d’Anapéou, arranged as karyotypes, are shown in Fig. 5k (plain, Giemsa stained) and Fig. 5l (C-banded). The arrangement of the chromosomes is the same as in Scottish *Boreonectes multilineatus* (Fig. 5i, j). The Pyrenean localities for *Boreonectes multilineatus* are shown in Fig. 9. Lac d’Anapéou is only about 25 km west of the previous locality from which it was recorded in the Pyrenees, Lac d’Oncet (Angus 2010b) but is on a different spur of the Pyrenees. Both of these localities are on the French side of the west-central Pyrenees. At the moment *Boreonectes ibericus* has not been found in the Pyrenees, but it may be expected to occur there as its range extends to the Alpes Maritimes (Dutton and Angus 2007).

### *Boreonectes ibericus* (Dutton & Angus, 2007)

An unbanded, Giemsa stained karyotype from a Lagos de Lloroza specimen is shown in Fig. 5u, while Fig. 5t shows one from a Peña Lara specimen. The Lloroza specimen is homozygous for the long (fused) form of autosome 1 and thus lacks any free autosome 24, while the Peña Lara specimen is heterozygous for the fusion and has one replicate of autosome 24. The Lagos de Lloroza (Fig. 9) are about 70 km further east than the Puerto de los Señales locality from where Dutton and Angus (2007) recorded *Boreonectes ibericus*, thus extending its known northern Spanish range a little closer to the Pyrenees.
